# An application of interactive fuzzy optimization model for redesigning supply chain for resilience

**DOI:** 10.1007/s10479-022-04542-5

**Published:** 2022-02-15

**Authors:** Kanokporn Kungwalsong, Abraham Mendoza, Vasanth Kamath, Subramanian Pazhani, Jose Antonio Marmolejo-Saucedo

**Affiliations:** 1 Graduate School of Management and Innovation, King Mongkut’s University of Technology Thonburi, Bangkok, Thailand; 2 Facultad de Ingeniería, Universidad Panamericana, Álvaro del Portillo 49, 45010 Zapopan, Jalisco Mexico; 3 Operations and Decision Sciences, T A Pai Management Institute, Manipal Academy of Higher Education, Manipal, 576104 India; 4 Advanced Analytics and Optimization Services Group, SAS Institute, 100 SAS Campus Dr, Cary, NC 27513 USA; 5 Facultad de Ingeniería, Universidad Panamericana, Augusto Rodin 498, 03920 Ciudad de México, Mexico

**Keywords:** Supply chain network design, Supply density, Disruption, Resilience, Interactive fuzzy optimization

## Abstract

Supply chain disruptions compel professionals all over the world to consider alternate strategies for addressing these issues and remaining profitable in the future. In this study, we considered a four-stage global supply chain and designed the network with the objectives of maximizing profit and minimizing disruption risk. We quantified and modeled disruption risk as a function of the geographic diversification of facilities called supply density (evaluated based on the interstage distance between nodes) to mitigate the risk caused by disruptions. Furthermore, we developed a bi-criteria mixed-integer linear programming model for designing the supply chain in order to maximize profit and supply density. We propose an interactive fuzzy optimization algorithm that generates efficient frontiers by systematically taking decision-maker inputs and solves the bi-criteria model problem in the context of a realistic example. We also conducted disruption analysis using a discrete set of disruption scenarios to determine the advantages of the network design from the bi-criteria model over the traditional profit maximization model. Our study demonstrates that the network design from the bi-criteria model has a 2% higher expected profit and a 2.2% lower profit variance under disruption than the traditional profit maximization solution. We envisage that this model will help firms evaluate the trade-offs between mitigation benefits and mitigation costs.

## Introduction

Increased competition and operating expenses have forced companies to strive toward cost optimization and increased service (Dai & Dai, 2016), allowing decision-makers (DMs) to extensively focus on supply chain (SC) operations (Ramezani et al., 2014; Ravindran & Warsing, 2016). Hence, SC network design decisions, which involve tasks such as selecting and partnering with the right suppliers (Araz et al., 2007; Xia & Wu, 2007), selecting facilities among potential locations (Turkoglu & Genevois, 2020), designing facility capacity (Irawan & Jones, 2019), and maximizing product flow between stages to achieve the best balance between investment and distribution cost under a set of given demands (Melo et al., 2009), have gained prominence. Sourcing has become easier because of improvements in information technology (IT) and transportation infrastructure. Such developments increase the complexity of SC functions and make them vulnerable to disruptions by mobilizing raw materials and finished goods from around the world (Pazhani & Ravindran, 2014).

Over the last decade, SC disruptions during the design of SC networks, particularly during supplier selection, have been widely studied (see Bilsel & Ravindran, 2011; Ravindran et al., 2010; Sawik, 2013, 2014). Several factors contribute to SC network disruption: *operational factors*, including equipment failure, electrical outages, unscheduled downtime, and road congestions; *political factors*, including terrorism, strikes, product recalls, and sudden changes in regulations; *environmental factors*, including severe weather, storms, floods, snow, landslides, and earthquakes; *strategic and control factors*, including just-in-time (JIT) and lean philosophy and low-cost off-shore sourcing strategies (Christopher et al., 2006); and *locational factors*, including complexity, node criticality, and density (Craighead et al., 2007). In this study, we considered the risk due to *locational factors* while designing an SC network under a disruption scenario.

Disruptions, particularly in SCs, expose the vulnerabilities of complex business systems around the world. Such disruptions occur not only upstream but also downstream, where hoarding and panic-buying consumer behavior cause equally significant disruptions to SCs (Nikolopoulos et al., 2020). Furthermore, the geographical clustering of facilities in the SC complicates this situation. Craighead et al. (2007) used the term supply chain density (SCD) to describe the geographical concentration of nodes within an SC in this context. They added that the severity of an SC disruption is directly related to the SCD. Hence, it is imperative to adopt flexibility in sourcing to mitigate disruptions due to locational factors (Snyder et al., 2016).

This study highlights the amalgamation of two theoretical areas to address the disruption problem. The first concept is systemic risk, which is derived from finance literature and is considered analogous to SC because different levels in both SCs and financial systems are interconnected (Scheibe & Blackhurst, 2018). This highlights the concept of contagion and propagation and explains how a small shock can cause havoc in systems (Elsinger et al., 2006). Keynes (1937) identified systemic risk during the Great Depression, and it has subsequently been used in fields other than economics, including climate and biology. This theory remains true in the case of complex SCs since a small shock in one part of the SC can propagate across the entire SC, causing havoc. This is further supplemented with the contingency theory (Burns & Stalker, 1961), which is widely used in organizational studies. According to this theory, an organization should optimize its performance by implementing a strategy that aligns its capabilities with environmental requirements (Mintzberg, 1978). These two theoretical foundations provide a lens for studying SC disruption. Since new trends such as lean, JIT, and other efficiency-focused activities are increasing, SCs are always at risk of potential disruptions. Hence, determining the impact of alternate SC designs on disruptions and SC profits is essential.

The SC network considered in this study is a well-known published network structure for real-world problems. The network structure is well-suited for a range of industries, including those related to consumer electronics, automotive, batteries, plastic goods, and glass. Based on the strategic nature of the problem, cost and resiliency are the main factors to consider while designing today’s global SCs. An SC disruption can cause a firm to have significant financial losses. This necessitates the search for the best SC design that can allow firms to operate cost-efficiently with the best network structure while remaining resilient to SC disruptions. With this motivation, the purpose of this study is to address the issue of designing a four-stage SC that considers profit and supply density. The problem involves determining the best suppliers, warehouse locations, and their capacity, as well as the distribution flow among the chosen facilities in the SC. Furthermore, the geographical dispersion of suppliers, which affects investment costs, product distribution, and redundancy, was considered in this study. According to our knowledge, there are few studies on SC network design models that explicitly integrate SCD characteristics, making it relevant in the current context and necessitating a separate study. We propose a bi-criteria mixed-integer linear programming (MILP) approach (Pazhani et al., 2018) for an SC network design problem with the dual objective of maximizing profit and SCD.

It is difficult to forecast and plan for demands for a strategic problem with long planning horizons. Firms may have to run the model with different input settings (e.g., different demand profiles) to finalize the best network structure. Furthermore, owing to the intrinsic multicriteria nature of the problem, firms would require an efficient algorithm to run the model and choose the best possible network structure from the available efficient solutions. This necessitates the search for and implementation of such an algorithm. An interactive fuzzy optimization algorithm is widely used to systematically solve the bi-criteria problem using the input of DMs. We adopted this algorithm and defined steps to guide users in this process. The flexibility of this methodology in analyzing and comparing scenarios helps firms to make high-quality, confident decisions. The model and proposed methodology are illustrated using a realistic example. Furthermore, the advantages of designing the SC while considering both cost and supply density are illustrated using disruption analysis. The analysis shows that the mitigation benefit outweighs the mitigation cost in the case of disruptions.

The remainder of this article is organized as follows. Section 2 reviews the literature on SC network design and disruption. Section 3 provides a detailed description of the SC network design problem for a four-stage SC with cost and supply density objectives and proposes a new bi-criteria MILP model for the problem. We also propose an interactive bi-criteria fuzzy optimization model to solve the bi-criteria model. Section 4 presents an example to illustrate the proposed bi-criteria mathematical model. Section 5 discusses the disruption scenario analysis to demonstrate the advantages of considering supply density to improve SC resiliency against disruptions. Section 6 presents the conclusions and future research directions.

## Literature review

Since the 1990s, competition in external businesses has encouraged organizations to improve efficiencies, and research on SCs has gained prominence as a result. Although SC network design has been studied extensively (Khalilpourazari & Arshadi Khamseh, 2019; Özceylan & Paksoy, 2013a, 2013b; Pervin et al., 2018; Sangaiah et al., 2020), awareness of the impact of disruptions in SC activities has recently increased.

SC disruption as a field of study is not new; it has coexisted with the SC field since its inception (Snyder et al., 2016). However, over the last two decades, the term “disruption” regarding SC has gained traction as follows:

- Several high-profile events, such as the terrorist attacks of September 11, 2001 (Stecke & Kumar, 2009), Hurricane Katrina in 2005 (Wachtendorf et al., 2013), and the more recent COVID-19 pandemic (Ivanov & Dolgui, 2020), have brought studies on SC disruptions to the forefront of public attention.
- According to Anderson (2007), McGillivray (2000), and Peck (2005), the JIT philosophy tends to intensify SC vulnerability during disruptions. This vulnerability is attributed to the marginal room for error, which is demanded by the inherent nature of a tightly optimized, lean design.
- As the vertical integration in supplier firms are decreasing, global SCs with suppliers all over the world are increasing (Cohen & Lee, 2020).

Furthermore, literature reviews conducted by contemporary researchers demonstrate the emergence of new dimensions to the SC design theme (Table 1).

**Table 1:** Studies on the relationship between SC characteristics and disruption

Author and year	Theme	Findings
Meixell and Gargeya (2005)	Reviewed 18 major research articles from 1982 to 2005 related to the decision support models for a global SC design	Addressed four aspects of modeling issues: decision variables in the model, performance metrics, SC integration, and globalization considerations
Melo et al. (2009)	Presented a literature review on 98 journal articles from 1998 to 2008 related to network design in SCs	Emphasized the importance of SC network design and how these decisions will have a long-lasting effect on a firm
Farahani et al. (2014)	Reviewed 135 peer-reviewed articles related to SC network design models, solution techniques, and applications	Focused on the effects of the competitive environment on SC network design
Snyder et al. (2016)	examined 180 studies organized into six categories: evaluating supply disruptions; strategic decisions; sourcing decisions; contracts and incentives; inventory; and facility location	the field is likely to continue to grow over the coming years, with seven areas identified as promising and important as avenues for future research

The field has evolved from simple decision support models in SC network design (Farahani et al., 2014; Meixell & Gargeya, 2005; Melo et al., 2009) to complex topics such as supply disruptions, sourcing decisions, and facility location (Snyder et al., 2016). For example, Fazli-Khalaf et al. (2017) proposed an effective hybrid robust fuzzy stochastic programming method to control parameter uncertainty and risk-aversion level in the context of a lead-acid battery SC case study. Özceylan and Paksoy (2013a) proposed a mixed-integer programming model for optimizing a general closed-loop SC network model with forward and reverse components. Khalilpourazari et al. (2020) used a neural-learning process to overcome new challenges based on past experiences. They considered three objective functions that minimized total transportation time and cost while minimizing unfulfilled demand in a real-world case in Iran. Goli and Aazami (2018) used an accelerated cuckoo optimization algorithm to optimize vehicle routing in a case study on dairy product distribution. Similarly, research on SC has demonstrated the use of algorithms and mathematical models in diverse scenarios.

Recent research has demonstrated that the field of supplier selection is expanding (Table 2). Some prominent examples are studies on framework development (De Boer et al., 2001), the need to focus on customer-oriented criteria (Ho et al., 2010), and the evaluation of uncertainty as a critical factor in supplier selection (Chai et al., 2013).

**Table 2:** Studies on the relationship between characteristics and disruption

Author and year	Theme	Findings
De Boer et al. (2001)	Studied the supplier selection literature in a more comprehensive manner	Proposed a framework that includes four main steps in the supplier selection process: problem definition, formulation of selection criteria, pre-qualification (preliminary screening), and final selection
Ho et al. (2010)	Presented a survey on 78 journal articles (between 2000 and 2008) related to multicriteria decision-making approaches for supplier evaluation and selection	Need to focus on customer-oriented criteria (quality, delivery, flexibility) instead of a cost-based approach to supplier selection
Chai et al. (2013)	Provided a literature review on 123 journal articles (from 2008 to 2012) on the application of decision-making techniques for supplier selection	Evaluating with the trend of uncertainty in supplier selection can be a promising direction for future studies

The literature reviews outlined in Tables 1 and 2 emphasize the importance of investigating uncertainty in SC designs. According to Sabri and Beamon (2000), uncertainty is one of the most challenging and prominent problems in SC management. The inherent stochastic nature of uncertainties is what makes a system complex, and it has attracted the interest of researchers all over the globe. For example, Goh et al. (2007) developed a stochastic model for a multistage global SC network problem. They used Moreau–Yosida regularization to design an algorithm for solving the multistage global SC network problem with the goals of maximizing profit and minimizing risk.

Similarly, Santoso et al. (2005) proposed a stochastic programming model and solution algorithm to solve a realistic SC network design problem. They combined the sample average approximation scheme with an accelerated Benders decomposition algorithm to solve large-scale stochastic SC design problems. Additionally, Salehi et al. (2017) adopted a new robust two-stage multiperiod stochastic model to design a blood supply network in Iran, taking into account the possibility of a natural disaster. They concluded that such studies are helpful in developing alternative strategies during SC disruptions.

SC disruption is one form of uncertainty that is garnering attention from practitioners and researchers because of increasing globalization (Ravindran et al., 2010) and instability in the system. Research has focused on both contextual and methodological contributions. Studies with contextual focus include disruption due to facility failures with equal (Snyder & Daskin, 2005) and unequal (Berman et al., 2007) probabilities, unreliable supplies (Qi & Shen, 2007), oligopolistic competition (Nagurney, 2010), facility disruptions (Peng et al., 2011), random demand and unreliable suppliers (Aryanezhad et al., 2012), and global pandemics (Chesbrough, 2020; Currie et al., 2020; Ivanov & Dolgui, 2020; Ivanov et al., 2018; Sarkis et al., 2020). Such low probability–high impact disruption effects can be mitigated by sourcing from nodes dispersed across the globe (Namdar et al., 2018).

The severity of SC disruptions is related to the geographical concentration of nodes within an SC, known as SCD, which results from an SC network design decision (Craighead et al., 2007; Falasca et al., 2008). SCD can be measured as the number of nodes divided by the average internode distance. The SCD is said to be high when many nodes are clustered within the SC. Therefore, the severity of SC disruptions is directly related to the SCD. Snyder et al. (2016) emphasized that disruption can be mitigated by sourcing flexibility, which can have an impact on overall SC profits. Thus, we focused on SCD and SC profits in this study.

Other studies have assessed the vulnerability of SCs and evaluated suitable mitigation strategies in response to uncertainty and disruption. Schmitt and Singh (2009) used the Monte Carlo analysis to generate a risk profile and discrete-event simulation to evaluate inventory policies suitable for distribution networks that consider demand uncertainty, supply uncertainty, or both. Klibi and Martel (2012) developed a scenario-based risk model to generate resilient SCs using the Monte Carlo analysis. In both studies, multiperiod risk profiles were generated to cover a specified planning horizon. Harrison et al. (2013) proposed an optimization approach called resiliency enhancement analysis via deletion and insertion (READI) to improve SC network resiliency. READI is used to evaluate network resiliency (when an important SC node or flow is disabled) and mitigation strategies for resilience improvements.

Empirical research has shown the relationship between SC network characteristics and SC disruption (Wagner & Neshat, 2010). SC network characteristics, including network decentralization, geographical dispersion, number of nodes, and number of tiers, appear to be related to the occurrence of SC disruptions (Bode & Wagner, 2015; Kim et al., 2015; Squire, 2010). Conversely, as shown in Table 3, characteristics such as density, complexity, node criticality, node centrality, and lack of redundancy are related to the severity of SC disruptions (Craighead et al., 2007; Falasca et al., 2008; Squire, 2010; Wagner & Bode, 2006).

**Table 3:** Studies on the relationship between SC characteristics and disruption

Authors	SC network characteristics	Focus
Wagner and Bode (2006)	Single sourcing and the reliance on global supply sources have a positive relationship to the severity of disruptions	Severity
Craighead et al. (2007) Falasca et al. (2008)	Density, complexity, node criticality, and capability of warning and recovery are positively related to the severity of disruptions	Severity
Squire (2010)	Node criticality and node centrality relate to the severity of the disruption Geographical distance relates to the probability of disruption Redundancy reduces the severity of the disruption Number of nodes relates to the probability of disruption	Severity Occurrence Severity Occurrence
Kim et al. (2015)	A network structure significantly relates to the likelihood of a network disruption	Occurrence
Bode and Wagner (2015)	The number of suppliers in each tier, the number of levels, and the geographical dispersion among members within the network have a positive relationship with the frequency of SC disruptions	Occurrence

It is noteworthy that most studies on SC design have focused on optimizing a single criterion, particularly profit (see Chan et al., 2016; Cheraghalipour et al., 2018; Darestani & Hemmati, 2019; Jiang et al., 2019; Latha Shankar et al., 2013; Sangaiah et al., 2020). However, DMs in many fields, including industry, engineering, and social sectors, are increasingly required to consider multiple conflicting objectives in their decision processes (Ravindran, 2016). Multicriteria decision-making problems are categorized based on whether the constraints are (i) finite and known or (ii) infinite and unknown (Ravindran, 2016). SC network design studies with multiple criteria have used a variety of solution methodologies, including variants of goal programming (Ravindran et al., 2010), Benders decomposition algorithm (Garcia-Herreros et al., 2014), exact mathematical modeling (Huang & Goetschalckx, 2014; Peng et al., 2011), and network optimization (Mari et al., 2014). Melo et al. (2009) provided a detailed review of location design in SCs.

We used multicriteria decision-making modeling (Pazhani et al., 2018; Pinto-Varela et al., 2011) to simulate the problem as a bi-criteria MILP in order to maximize profit and supply density. The interstage distance between SC nodes was used to calculate and maximize supply density, which resulted in a geographically dispersed network design. Subsequently, we propose an interactive fuzzy optimization algorithm that uses the *ɛ*-constraint method to solve the problem and generate a Pareto-efficient frontier. The interactive optimization algorithm guides the user/DM in choosing the best network design solution from those available in the Pareto-efficient frontier, based on the firm’s objectives. The model is illustrated using a realistic example. We also evaluated the resilience of the SC network solutions under disruptive scenarios and demonstrated the value of incorporating supply density into the network design. This research contributes to the existing literature by incorporating SC characteristics into SC design. Additionally, the proposed interactive optimization algorithm systematically solves the bi-criteria problem. The algorithm reduces the cognitive burden on the DM by accelerating the convergence of the best compromise solution.

## Bi-criteria network design model

**Fig. 1:**
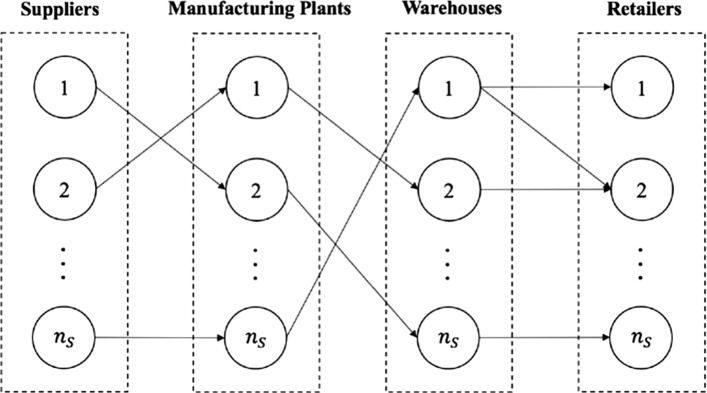
The four-stage SC network

This section presents the proposed bi-criteria network design model for the four-stage SC network with the objectives of maximizing profit and supply density. Let *S* = {1, 2,…, *n*_*S*_} be the set of suppliers, *M* = {1, 2,…, *n*_*M*_} the set of manufacturing plants, and *C* = {1, 2,…, *n*_*C*_} the set of retailers. Let *W* = {1, 2,…, *n*_*W*_} be the set of potential warehouse sites and *L* = {1,2,…, *n*_*L*_} the set of the warehouse capacity levels. Figure 1 shows the considered SC network.

The SC network includes suppliers, manufacturing plants, warehouses, and retailers. The manufacturing plants procure the raw material required for production from the set of selected suppliers. The product flows from the manufacturing plants to the warehouses, which are managed by a single DM (i.e., a centralized control system). The retailers are faced with demands from customers and are supplied by a set of potential warehouses.

The proposed model is developed to (i) select the appropriate suppliers and determine the quantities allocated to the chosen suppliers, (ii) select the appropriate set of warehouses and their capacity levels to distribute products from the manufacturing plants to retailers, and (iii) determine the flow of products in the SC between the selected set of facilities. The model’s objective is to maximize SC profit and supply density. The total cost includes purchasing costs, manufacturing costs, warehouse opening costs, and transportation costs between SC stages. The supply density objective was calculated based on the interstage distances between the selected set of suppliers and the manufacturing plants to which they supply raw materials and the intrastage distances between the selected group of suppliers. Hence, a lower supply density value implies that the nodes in the SC are clustered. Thus, we maximized the supply density.

The model parameters, decision variables, and cost function components are provided below:

- **Input Parameters**

**Table:** 

*cap*_*m*_	Production capacity at the manufacturing plant *m*, $\forall \;{\text{m}} \in M$
$cap_{w}^{l}$	Capacity of warehouse w of size *l*, $\forall \;{\text{w}} \in W,\;\forall \;{\text{l}}\; \in L$
*cap*_*s*_	Capacity of supplier *s*, $\forall \;{\text{s}} \in S$
*d*_*c*_	Demand for products at retailer *c*, $\forall \;{\text{c}} \in C$
*dis*_*sm*_	Distance between supplier s and manufacturing plant *m*, $\forall \;{\text{s}} \in S,\;\forall \;{\text{m}}\; \in M$
*idis*_*ss’*_	Intrastage distance between suppliers *s* and *s*’, $\forall \;{\text{s}},\;{\text{s}}^{^{\prime}} \in S$
*msm*	Minimum transportation quantity from suppliers to manufacturers
*mas*	Maximum number of suppliers to be selected in the network

- **Cost function components**

**Table:** 

*p*_*sm*_	Purchasing cost of raw materials from supplier *s* by plant *m*, $\forall \;{\text{s}} \in S,\;\forall \;{\text{m}}\; \in M$
*tr*_*mw*_	Transportation cost per unit from plant *m* to warehouse *w*, $\forall \;{\text{m}} \in M,\;\forall \;{\text{w}}\; \in W$
*tr*_*wc*_	Transportation cost per unit from warehouse *w* to retailer *c*, $\forall \;{\text{w}} \in W,\;\forall \;{\text{c}}\; \in C$
*pc*_*m*_	Production cost for a product at plant *m*, $\forall \;{\text{m}} \in M$
*np*	Price of a product
$f_{w}^{l}$	Fixed cost of opening warehouse *w* with capacity *l*, $\forall \;{\text{w}} \in W,\;\forall \;{\text{l}}\; \in L$
*ls*_*c*_	Lost sales cost at retailer *c*, $\forall \;{\text{c}}\; \in C$

- **Decision variables**

**Table:** 

*QSM*_*sm*_	Quantity of raw materials purchased from supplier *s* by plant *m*, $\forall \;{\text{s}} \in S,\;\forall \;{\text{m}}\; \in M$
*QMW*_*mw*_	Quantity of products transported from plant *m* to warehouse *w*, $\forall \;{\text{m}} \in M,\;\forall \;{\text{w}}\; \in W$
*QWC*_*wc*_	Quantity of products transported from warehouse *w* to retailer *c*, $\forall \;{\text{w}} \in W,\;\forall \;{\text{c}}\; \in C$
*LD*_*c*_	Quantity of sales lost at retailer *c*, $\forall \;{\text{c}} \in C$
$\delta_{w}^{l}$	$$\left\{ {\begin{array}{*{20}l} {{\text{1, if warehouse}} w{\text{ is opened with size }}l} \\ {\text{0, otherwise }} \\ \end{array} } \right., \forall w \in W,l \in L$$
*Sα*_*sm*_	$$\left\{ {\begin{array}{*{20}l} {{\text{1, if supplier}} s{\text{ supplies raw materials to plant }}m} \\ {\text{0, otherwise }} \\ \end{array} } \right., \forall s \in S,m \in M$$
*Sβ*_*ijm*_	$$\left\{ {\begin{array}{*{20}l} {{\text{1, if supplier}} i{\text{ and supplier }}j{\text{ supply to plant }}m} \\ {\text{0, otherwise }} \\ \end{array} } \right., \forall \left( {i,j} \right) \in S{\text{ and }}\left( {i \ne j} \right),m \in M$$
*Sβ’*_*ijm*_	$$\left\{ {\begin{array}{*{20}l} {{\text{1, if supplier}} i{\text{ and supplier }}j{\text{ supply to plant }}m} \\ {\text{0, otherwise }} \\ \end{array} } \right., \forall \left( {i,j} \right) \in S{\text{ and }}\left( {i \ne j} \right),m \in M$$
*SUP*_*s*_	$$\left\{ {\begin{array}{*{20}l} {{\text{1, if supplier}} s{\text{ supplies raw materials}}} \\ {\text{0, otherwise }} \\ \end{array} } \right., \forall s \in S$$

The following assumptions were considered when developing the proposed model:

(i) Retailer demands are deterministic. Since the proposed model is a strategic decision-making model, this assumption is reasonable.
(ii) The suppliers and manufacturing plants have finite production capacity.
(iii) The cost of transporting raw materials from the supplier to the manufacturing plant is included in the raw material purchasing cost.
(iv) The warehousing facilities in the SC have capacity restrictions.

### The proposed bi-criteria MILP model

Considering the purchasing cost, manufacturing cost, transportation cost, fixed cost components, interdistance between the suppliers and manufacturing plants, and intradistance between the suppliers, the problem can be formulated as an MILP with the objectives of maximizing the total profit and the supply density of the SC:

#### Objective 1: maximizing the SC profit (***Z***_1_)

$$ \begin{aligned} {\text{Maximize}}\quad Z_{1} = & \left\{ {np\left( {\mathop \sum \limits_{w \in W} \mathop \sum \limits_{c \in C} QWC_{wc} } \right)} \right\} - \left\{ {\mathop \sum \limits_{s \in S} \mathop \sum \limits_{m \in M} p_{sm} QSM_{sm} } \right. \\ & + \mathop \sum \limits_{m \in M} pc_{m} \left( {\mathop \sum \limits_{w \in W} QMW_{mw} } \right) + \mathop \sum \limits_{m \in M} \mathop \sum \limits_{w \in W} tr_{mw} QMW_{mw} \\ & \left. { + \mathop \sum \limits_{w \in W} \mathop \sum \limits_{c \in C} tr_{wc} QWC_{wc} + \mathop \sum \limits_{l \in L} \mathop \sum \limits_{w \in W} f_{w}^{l} \delta_{w}^{l} + \mathop \sum \limits_{c \in C} ls_{c} LD_{c} } \right\}, \\ \end{aligned} $$

where the components of the SC profit objective are: {revenue}—{purchasing cost + production cost + transportation cost from plants to warehouses + transportation cost from warehouses to retailers + fixed cost for opening warehouses + lost sales cost}.

#### Objective 2: maximizing the supply density based on the interstage distance (***Z***_2_)

**Fig. 2:**
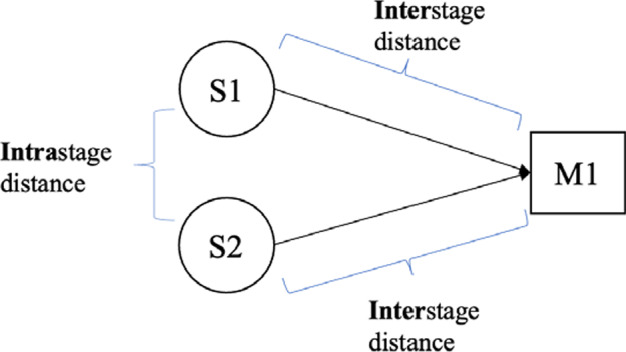
Interstage distance and intrastage distance

The supply density is calculated based on the interstage distance between the selected suppliers and plants and the intrastage distance between the selected suppliers. We will demonstrate the computation with an example. Consider a two-stage SC where two suppliers (S1 and S3) both supply products to a manufacturer (M1). The interstage distance is the distance between stages. In this example, the interstage distance is the sum of the distance between supplier S1 and manufacturer M1 and the distance between supplier S3 and manufacturer M1 (see Fig. 2). The interstage density of the suppliers in the SC is calculated using this metric. The intrastage distance is the distance within a stage. In this example, the intrastage distance is the distance between suppliers S1 and S3, as shown in Fig. 2. The intrastage density of suppliers within a stage in the SC is measured using this metric.

In this study, the supply density objective is the density of the supply entity per unit of demand, which is defined by the interstage and intrastage distances for the supplier stage in the objective function:

$$ {\text{Maximize}}\quad Z_{2} = \frac{1}{{\mathop \sum \nolimits_{c \in C} d_{c} }}\left( {\mathop \sum \limits_{s \in S} \mathop \sum \limits_{m \in M} dis_{sm} S\alpha_{sm} + \mathop \sum \limits_{m \in M} \mathop \sum \limits_{i \in S} \mathop \sum \limits_{j \in S} dis_{ij} S\beta_{ijm} } \right) $$

Subject to,

1 $$ \mathop \sum \limits_{m \in M} QSM_{sm} \le cap_{s} , \forall s \in S, $$

2 $$ QSM_{sm} \le cap_{s} \times S\alpha_{sm} ,\forall s \in S, \forall m \in M $$

3 $$ QSM_{sn} \ge msm \times S\alpha_{sm} , \forall s \in S, \forall m \in M $$

4 $$ \left( {2 \times S\beta_{ijm} } \right) + S\beta^{\prime}_{ijm} = S\alpha_{im} + S\alpha_{jm} , \forall \left( {i,j} \right) \in S, i \ne j,\quad {\text{i}} < {\text{j}},\;\forall m \in M $$

5 $$ S\beta_{ijm} + S\beta^{\prime}_{ijm} \le 1, \forall \left( {i,j} \right) \in S, i \ne j, i < j, \forall m \in M $$

6 $$ \mathop \sum \limits_{w \in W} QMW_{mw} \le cap_{m} , \forall m \in M $$

7 $$ \mathop \sum \limits_{s \in S} QSM_{sm} = \mathop \sum \limits_{w \in W} QMW_{mw} , \forall m \in M $$

8 $$ \mathop \sum \limits_{m \in M} QMW_{mw} \le \mathop \sum \limits_{l \in L} cap_{w}^{l} \times \delta_{w}^{l} , \forall w \in W $$

9 $$ \mathop \sum \limits_{l \in L} \delta_{w}^{l} \le 1, \forall w \in W $$

10 $$ \mathop \sum \limits_{m \in M} QMW_{mw} \le \mathop \sum \limits_{c \in C} QWC_{wc} , \forall w \in W $$

11 $$ \mathop \sum \limits_{w \in W} QWC_{wc} + LD_{c} = d_{c} , \forall c \in C $$

12 $$ \mathop \sum \limits_{m \in M} S\alpha_{sm} \le M \times SUP_{s} , \forall s \in S $$

13 $$ \mathop \sum \limits_{s \in S} SUP_{s} \le mas $$

14 $$ QSM_{sm} , QMW_{mw} ,QWC_{wc} , LD_{c} \ge 0 $$

15 $$ \delta_{w}^{l} , S\alpha_{sm} , S\beta_{ijm} , S\beta_{ijm }^{^{\prime}} \in \left\{ {0.1} \right\} $$

Each supplier *s* has a finite supply capacity: *cap*_*s*_. Constraint set (1) ensures that the quantity of raw materials supplied by supplier *s* to all manufacturing plants is less than or equal to its capacity. Constraint sets (2) and (3) determine the binary variables for the interstage flow between suppliers and manufacturers. Constraint (2) ensures that if there is a shipment between supplier *s* and manufacturer *m*, the binary variable *Sα*_*sm*_ = 1. Constraint (3) ensures that if there is no shipment between stages, the binary variable *Sα*_*sm*_ = 0. Constraint (3) also ensures minimum shipment if there is a shipment between supplier *s* and plant *m.* Constraint sets (4) and (5) determine the binary variables for the intrastage flow for the supplier stage. The right-side term in Constraint (4) represents the product flow from suppliers *i* and *j* to manufacturer *m*. If both (supplier *i* to manufacturer *m* and supplier *j* to manufacturer *m*) links have product flow, the binary variable *Sβ*_*ijm*_ = 1 and *Sβ’*_*ijm*_ = 0. If product flow exists in either the supplier *i* to manufacturer *m* link or the supplier *j* to manufacturer *m* link, then *Sβ’*_*ijm*_ = 1 and *Sβ*_*ijm*_ = 0. If there is no flow in these links, *Sβ*_*ijm*_ = 0 and *Sβ’*_*ijm*_ = 0. Constraint (5) ensures that one of the following cases is true: *Sβ*_*ijm*_ = 1 and *Sβ’*_*ijm*_ = 0, *Sβ*_*ijm*_ = 0 and *Sβ’*_*ijm*_ = 1, or *Sβ*_*ijm*_ = 0 and *Sβ’*_*ijm*_ = 0. Constraint (6) is the production capacity constraints at the plants. The left-most term represents the total quantity of products transported to warehouses from plant *m*, which should be less than or equal to its capacity.

Constraint set (7) ensures that the quantity of raw materials flowing into plant *m* equals the number of products flowing out of the plant to the warehouses. Constraint set (8) ensures that if a warehouse is selected, the number of products flowing into the warehouse, *w*, does not exceed the storage capacity. The left side represents the total quantity of products flowing into warehouse *w*. The right side is the capacity of the selected warehouse. If warehouse *w* is opened, constraint set (9) ensures that only one capacity level is preferred. Constraint set (10) ensures that the quantity of products flowing into warehouse *w* equals the number of new products flowing out of the warehouse to the retailers. Constraint set (11) represents the demand satisfaction constraints. The total quantity of products flowing into retailer *c* and the lost sales at retailer *c* should be equal to the demand at that retailer.

Constraint set (12) ensures that the variable, *SUP*_*s*_, is set to 1 when there is a flow from supplier *s*. Note that *M* is a large positive number*.* Constraint (13) also ensures that the total number of selected suppliers does not exceed the maximum. Finally, Constraints (14) and (15) describe the nonnegativity and binary conditions of the decision variables.

### Interactive fuzzy optimization algorithm

Fuzzy programming methods are popular approaches for solving multiobjective programming models because of their ability to explicitly measure and adjust the satisfaction level of each objective function (Pishvaee & Razmi, 2012). Herein, we propose a fuzzy solution method based on the *ɛ*-constraint method (Hwang & Masud, 1979). This method provides DMs an appropriate picture of the entire Pareto-optimal set, allowing them to select their preferred solution. The advantage of this method is that after the entire Pareto-optimal set has been defined, the DM can determine the final decision more confidently based on comprehensive available information (Pishvaee & Razmi, 2012; for a detailed description of the *ɛ*-constraint method, see Ehrgott, 2005).

We first generate a set of efficient solutions by varying the right-hand side of the *ɛ*-constraint. The *ɛ* values are first varied in more comprehensive steps to develop the entire Pareto-optimal solution. Next, we present the efficient frontier to the DM, and the DM chooses a range of *ɛ* values that they are interested in. We use the DM’s input to generate solutions in the interested range by varying the *ɛ* values in more adequate steps. Subsequently, the DM is presented with this solution to determine the best compromise solution. We used an interactive fuzzy optimization algorithm based on the *ɛ*-constraint method for solving the bi-criteria MILP model.

The general form of the bi-criteria mathematical programming model is as follows:

$$ {\text{Maximize}}\quad f_{1} \left( x \right), $$

$$ {\text{Maximize}}\quad f_{2} \left( x \right), $$

$$ {\text{Subject to}},\quad y_{i} \left( x \right) \le 0,\forall \left( {1 \le i \le m} \right), $$

where *x* is an *n*-dimensional vector of the decision variables, *f*_1_ and *f*_2_ represent the profit and density objectives, respectively, and *y*_*i*_ represents Constraint sets (1) to (15).

Let ${\text{S}} = \left\{ {x \left| {y_{i} \left( x \right) \le 0} \right.} \right\}$ denote the feasible region. We aimed to find the best compromise solution that maximizes profit and SCD based on the DMs’ utility function. The steps of the proposed method are as follows:
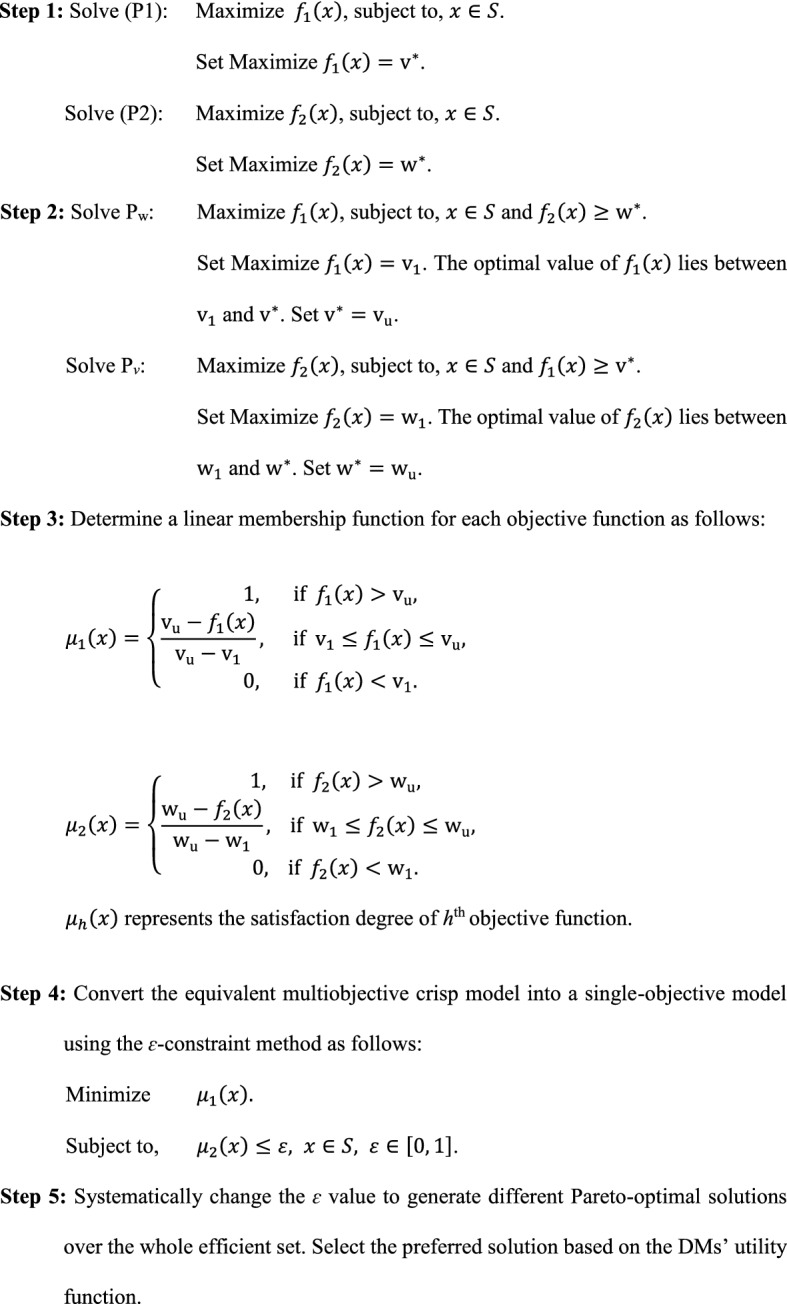


At each iteration, we minimized $\mu_{1} \left( x \right)$ with constraint on $\mu_{2} \left( x \right)$. The problem can also be solved by minimizing $\mu_{2} \left( x \right)$ with a constraint on $\mu_{1} \left( x \right)$.

## Illustrative example

This section presents an analysis of a four-stage SC system as an example. The SC network comprises the following:

- Twenty potential suppliers of materials required to manufacture new products,
- Five manufacturing plants that produce new products and refurbish returned products,
- Twenty-five possible warehousing facilities for distributing new products to retailers, and
- Approximately 100 retailers, who face demand from customers.

The input and cost-parameter settings used in this example are more realistic with respect to the location of the facilities and the distances between them. The SC facilities are geographically spread across six different regions around the world (see Table 4). We selected latitude and longitude for each facility in the SC. Thereafter, we used the FINDDIST procedure of Ramkumar et al. (2012) to determine the interstage and intrastage distances between the facilities. See "Appendix" for Tables 18 and 19: Table 18 shows the interstage distance between the suppliers and manufacturers, and Table 19 shows the intrastage distance between the suppliers. Note that the intrastage distance between suppliers *i* and *j*, where *i* = *j* is not feasible, is assigned a significant value of 100,000.

**Table 4:** Geographical locations of existing and potential facilities

Region	Suppliers	Plants	Warehouses	Retailers
Region 1 (Africa)	S16	M3	W17	R65–R68
Region 2 (Asia)	S5, S8, S10, S11, S12, S15, S17, S18	M1, M2	W7, W9, W10, W11, W14, W15, W16, W19, W20, W21, W25	R25–R28, R33–R44, R53–R64, R73–R84, R97–R100
Region 3 (Europe)	S6, S7, S9, S14, S19	M4	W3, W4, W8, W13, W18, W22	R9–R16, R29–R32, R49–R52, R69–R72, R85–R88
Region 4 (North America)	S4, S13, S20	M5	W6, W12, W23	R21–R24, R45–R48, R89–R92
Region 5 (Australia)	S2	–	W2	R5–8
Region 6 (South America)	S1, S3	–	W1, W5, W24	R1–R4, R17–R20, R93–R96
Total facilities	20	5	25	100

The cost parameters are modeled as a function of the product price. Table 5 shows the cost settings. Note that the purchasing cost of the raw materials includes the distances between the supplier and the manufacturing plants (*dis*_*sm*_).

**Table 5:** Cost-parameter settings for the numerical example

Parameter	Notation	Setting
Total cost of a new product	*pp*	$750
Profit margin		20%
Price of new product	*np*	$900
Purchasing cost of raw material	*p*_*sm*_	~ Unif (60%, 65%) * *pp* + (*dis*_*sm*_ / 250)
Production cost for a new product	*pc*_*m*_	~ Unif (8%, 12%) * *pp*
Transportation cost per unit between plant and warehouse	*tr*_*mw*_	~ Unif (5.5%, 6.5%) * *pp*
Transportation cost per unit between retailer and warehouse	*tr*_*wc*_	~ Unif (8.5%, 9.5%) * *pp*

Retailer demand was generated from a uniform distribution between 500 and 700 units. The maximum number of suppliers (*mas*) to be selected was set as 10. Table 20 (see "Appendix") shows the purchasing cost and capacity of the suppliers, as well as the capacity of the manufacturing plants. Table 21 (see "Appendix") shows the capacity of the warehouses and fixed costs.

We then solved and analyzed the proposed bi-criteria model and the interactive solution method using the illustrative example. The example was coded in Microsoft Visual C++ 6.0 and solved using ILOG Concert Technology with CPLEX 12.1 on a personal computer with a 2.8 GHz INTEL(R) Core (TM) 2 Duo Processor and 2.0 GB RAM. We first ran the model as a single-objective problem with *profit* (*Z*_1_) and *density* (*Z*_2_) as separate objectives. Thereafter, we ran the model as a bi-criteria model with both *profit* (*Z*_1_) and *density* (*Z*_2_) objectives using the proposed interactive fuzzy optimization algorithm.

### Single-objective model solutions

**Fig. 3:**
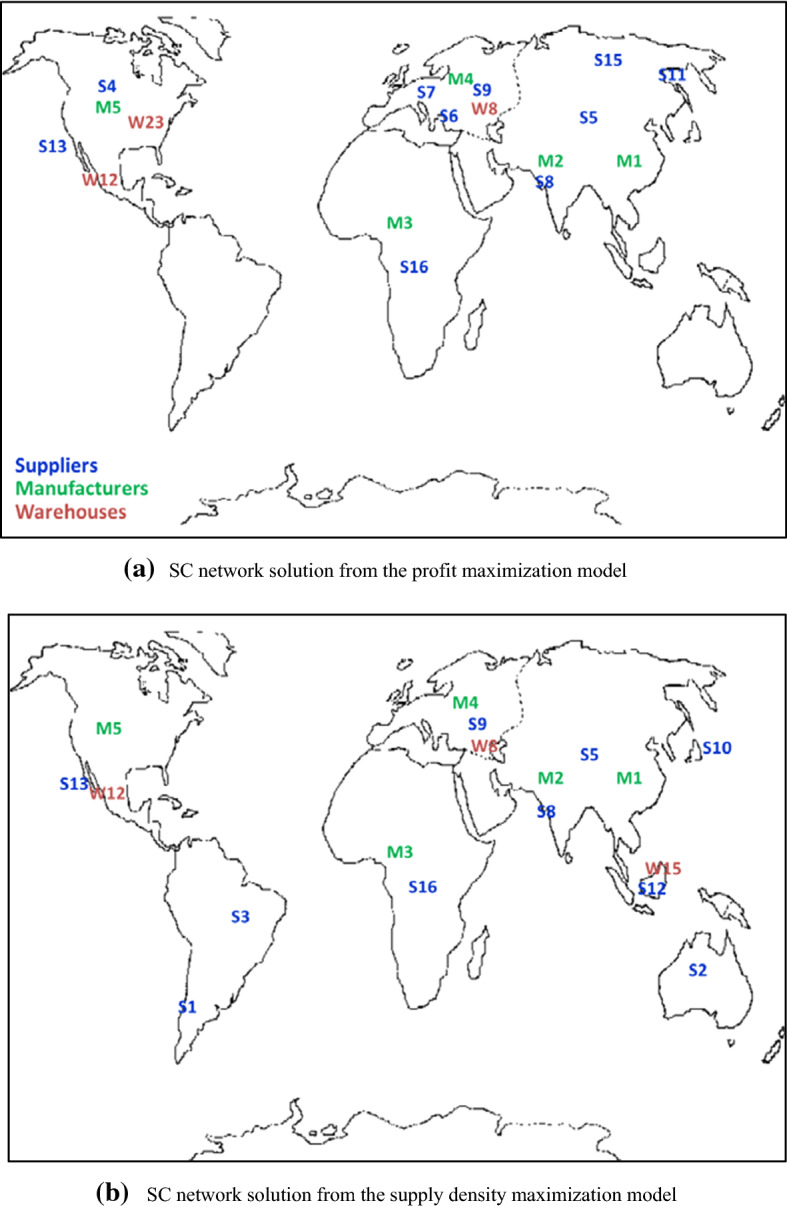
Geographical dispersion of the SC network from the single-objective model

We started by solving the single-objective models to obtain the ideal value of the objective functions. For the profit maximization model, the ideal value of SC profit (v_u_) was $13,160,455.48, with a supply density (w_l_) of 0.81. For the supply density maximization model, the ideal value of the supply density (w_u_) was 30.01, with an SC profit (v_l_) of $11,257,836.05. Table 6 summarizes the network design from the single-objective models. Figure 3 presents the geographical dispersion of the SC network solutions.

**Table 6:** Single-objective model solutions

Region	Profit maximization		Supply density maximization	
	Selected suppliers	Selected warehouses	Selected suppliers	Selected warehouses
Region 1	S16	–	S16	–
Region 2	S5, S8, S11, S15	–	S5, S8, S10, S12	W15
Region 3	S6, S7, S9	W8	S9	W8
Region 4	S4, S13	W12, W23	S13	W12
Region 5	–	–	S2	–
Region 6	–	–	S1, S3	–
Profit value	$13,160,455.48 (ideal profit value)		$11,257,836.05	
Density value	0.81		30.01 (ideal density value)

### Bi-criteria model solutions

**Fig. 4:**
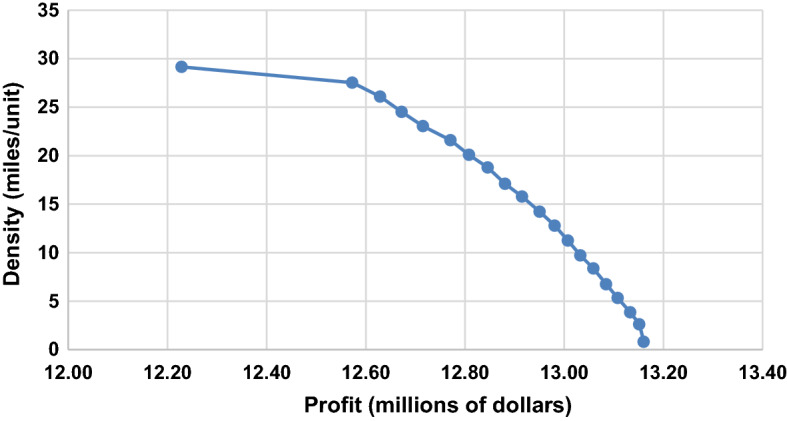
The efficient frontier for *ɛ* values between 0 and 1

In this section, we solve the bi-criteria model with profit and density objectives using the proposed interactive fuzzy optimization algorithm based on the *ɛ*-constraint method. Pishvaee and Razmi (2012) suggested that the DM could adjust the range of the *ɛ* values throughout the calculation process. In early iterations, the DM starts with more comprehensive steps to quickly generate the whole range of Pareto-optimal solutions. In later iterations, the DM may be interested in selecting the final preferred solution using finer steps such that interesting areas can be investigated more precisely. Initially, we vary the *ɛ* values between 0 and 1 in steps of 0.05. Table 7 shows the revenue, costs, SC profit, and supply density for *ɛ* values ranging from 0 to 1. Figure 4 shows the entire efficient frontier.

**Table 7:** Summary of the results for *ɛ* values between 0 and 1

$$\varepsilon$$	CPU time (secs)	μ_1_	μ_2_	Revenue ($)	Purchasing cost ($)	Production cost ($)	Transportation cost ($)	Fixed cost ($)	Lost sales cost ($)	Profit ($)	Density (miles/unit)
0.00	881.22	0.50	0.50	49,149,000.00	26,161,019.81	4,074,865.68	5,869,694.47	1,668,309.00	117,275.00	11,257,836.05	30.01
0.05	596.61	0.49	0.04	52,496,100.00	27,843,260.62	4,392,492.22	6,271,701.96	1,730,826.00	28,943.00	12,228,876.20	29.15
0.10	152.39	0.31	0.10	53,607,600.00	28,395,758.01	4,477,201.70	6,406,662.02	1,755,097.00	–	12,572,881.27	27.52
0.15	138.73	0.28	0.15	53,554,500.00	28,328,484.62	4,459,104.26	6,387,588.84	1,749,042.00	1298.00	12,628,982.28	26.08
0.20	468.85	0.26	0.20	53,607,600.00	28,309,669.49	4,462,117.16	6,408,088.09	1,755,097.00	–	12,672,628.26	24.50
0.25	1208.73	0.23	0.25	53,607,600.00	28,270,286.14	4,463,281.80	6,403,502.06	1,755,097.00	–	12,715,433.00	23.04
0.30	245.44	0.20	0.30	53,554,500.00	28,208,471.38	4,440,798.12	6,383,964.70	1,749,042.00	1298.00	12,770,925.80	21.59
0.35	1030.15	0.19	0.35	53,554,500.00	28,171,409.20	4,441,443.90	6,383,118.69	1,749,042.00	1298.00	12,808,188.21	20.08
0.40	338.15	0.17	0.39	53,607,600.00	28,175,613.76	4,436,588.13	6,391,485.55	1,758,001.00	–	12,845,911.56	18.78
0.45	388.94	0.15	0.45	53,554,500.00	28,085,589.94	4,449,868.27	6,387,862.04	1,749,042.00	1298.00	12,880,839.75	17.10
0.50	150.18	0.13	0.49	53,554,500.00	28,058,530.71	4,444,584.86	6,386,001.77	1,749,042.00	1298.00	12,915,042.66	15.78
0.55	156.08	0.11	0.55	53,554,500.00	28,016,040.52	4,449,911.55	6,387,545.84	1,749,042.00	1298.00	12,950,662.09	14.22
0.60	133.14	0.09	0.60	53,554,500.00	27,982,413.98	4,454,135.99	6,386,592.78	1,749,042.00	1298.00	12,981,017.25	12.78
0.65	140.33	0.08	0.65	53,554,500.00	27,951,818.28	4,457,005.40	6,387,795.36	1,749,042.00	1298.00	13,007,540.96	11.24
0.70	194.56	0.07	0.70	53,554,500.00	27,930,002.62	4,453,426.74	6,387,902.42	1,749,042.00	1298.00	13,032,828.22	9.71
0.75	88.69	0.05	0.75	53,554,500.00	27,906,146.83	4,450,986.10	6,387,975.43	1,749,042.00	1298.00	13,059,051.64	8.36
0.80	94.76	0.04	0.80	53,554,500.00	27,878,138.51	4,453,423.57	6,387,925.60	1,749,042.00	1298.00	13,084,672.32	6.74
0.85	91.19	0.03	0.85	53,554,500.00	27,851,167.73	4,456,356.44	6,388,664.55	1,749,042.00	1298.00	13,107,971.28	5.32
0.90	109.22	0.01	0.90	53,554,500.00	27,818,302.30	4,462,804.50	6,389,760.07	1,749,042.00	1298.00	13,133,293.13	3.85
0.95	89.59	0.00	0.94	53,554,500.00	27,800,202.15	4,462,222.18	6,390,142.45	1,749,042.00	1298.00	13,151,593.22	2.61
1.00	7.49	0.50	0.50	53,554,500.00	27,784,596.65	4,468,797.16	6,390,310.71	1,749,042.00	1298.00	13,160,455.48	0.81

**Fig. 5:**
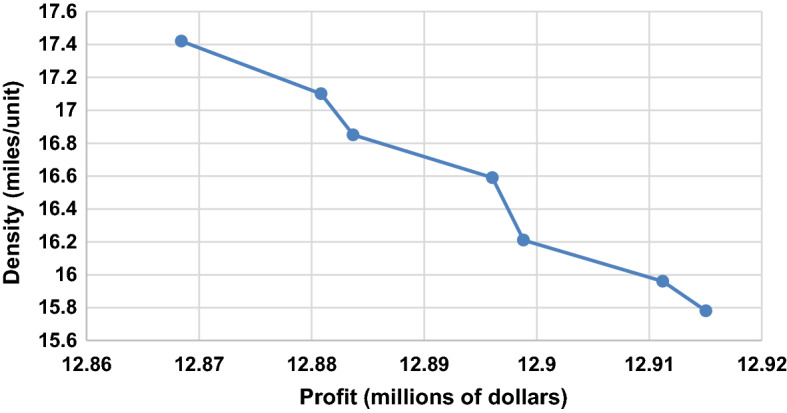
The efficient frontier for *ɛ* values between 0.44 and 0.50

We present the DM with this efficient frontier solution. We assume that the DM selects an *ɛ* value range of 0.45–0.50. We generate the Pareto-optimal solutions using a finer step value of 0.01. Table 8 shows the corresponding results, and Fig. 5 shows the graphical representation of the efficient frontier.

**Table 8:** Summary of the results for *ɛ* values between 0.44 and 0.50

$$\varepsilon$$	CPU time (secs)	μ_1_	μ_2_	Revenue ($)	Purchasing cost ($)	Production cost ($)	Transportation cost ($)	Fixed cost ($)	Lost sales cost ($)	Profit ($)	Density (miles/unit)
0.44	186.48	0.15	0.44	53,554,500.00	28,101,635.18	4,446,970.56	6,387,126.66	1,749,042.00	1,298.00	12,868,427.60	17.42
0.45	293.60	0.15	0.45	53,554,500.00	28,085,589.94	4,449,868.27	6,387,862.04	1,749,042.00	1,298.00	12,880,839.80	17.10
0.46	146.72	0.15	0.46	53,554,500.00	28,093,715.04	4,441,104.82	6,385,648.76	1,749,042.00	1,298.00	12,883,691.40	16.85
0.47	197.33	0.14	0.47	53,554,500.00	28,074,036.98	4,446,935.41	6,387,123.09	1,749,042.00	1,298.00	12,896,064.50	16.59
0.48	143.24	0.14	0.48	53,554,500.00	28,078,773.96	4,440,522.50	6,386,031.13	1,749,042.00	1,298.00	12,898,832.40	16.21
0.49	384.10	0.13	0.49	53,554,500.00	28,059,095.90	4,446,353.08	6,387,505.46	1,749,042.00	1,298.00	12,911,205.60	15.96
0.50	150.18	0.13	0.49	53,554,500.00	28,058,530.71	4,444,584.86	6,386,001.77	1,749,042.00	1,298.00	12,915,042.70	15.78

**Fig. 6:**
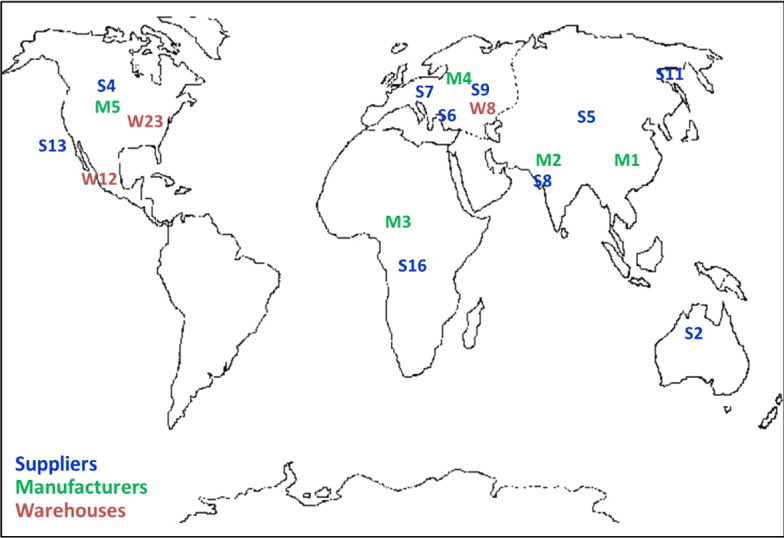
SC network configuration for the bi-criteria model

We assume that the DM selects the efficient solution for *ɛ* = 0.45. Table 9 presents the selected suppliers and warehouses from the bi-criteria model with an *ɛ* value of 0.45. Suppliers from five regions are selected. Table 10 shows the material flows across the SC stages. Three warehouses (warehouses 8, 12, and 23), each with a size of 3, are selected to distribute the finished products to the retailers. The product flows between the manufacturing plants, and the selected warehouses are presented in Table 11. Figure 6 shows the SC network configuration from the bi-criteria model for *ɛ* = 0.45.

**Table 9:** Selected suppliers and warehouses from the bi-criteria model solution

Region	Selected suppliers	Selected warehouses
Region 1	S16	–
Region 2	S5, S8, S11	–
Region 3	S6, S7, S9	W8
Region 4	S4, S13	W12, W23
Region 5	S2	–
Region 6	–	–

**Table 10:** Product flows from the selected suppliers to the manufacturing plants

Facilities	Manufacturing plant 1	Manufacturing plant 2	Manufacturing plant 3	Manufacturing plant 4	Manufacturing plant 5	Total
Supplier 1	0	0	0	0	0	0
Supplier 2	500	2488	500	500	500	4488
Supplier 3	0	0	0	0	0	0
Supplier 4	500	500	500	500	4295	6295
Supplier 5	3975	500	500	500	500	5975
Supplier 6	500	500	500	3788	500	5788
Supplier 7	0	500	891	4741	500	6632
Supplier 8	0	6382	500	0	0	6882
Supplier 9	0	500	5990	500	0	6990
Supplier 10	0	0	0	0	0	0
Supplier 11	2170	500	500	0	500	3670
Supplier 12	0	0	0	0	0	0
Supplier 13	0	500	500	500	4688	6188
Supplier 14	0	0	0	0	0	0
Supplier 15	0	0	0	0	0	0
Supplier 16	0	500	6097	0	0	6597
Supplier 17	0	0	0	0	0	0
Supplier 18	0	0	0	0	0	0
Supplier 19	0	0	0	0	0	0
Supplier 20	0	0	0	0	0	0
Total	7645	12,870	16,478	11,029	11,483	59,505

**Table 11:** Product flows from the manufacturing plants to the selected warehouses

Facilities	Warehouse 8	Warehouse 12	Warehouse 23	Total
Manufacturing plant 1	3143	0	4502	7645
Manufacturing plant 2	0	0	12,870	12,870
Manufacturing plant 3	16,478	0	0	16,478
Manufacturing plant 4	0	9724	1305	11,029
Manufacturing plant 5	0	11,483	0	11,483
Total	19,621	21,207	18,677	59,505

## Disruption analysis

In this section, we present the disruption analysis using a discrete set of scenarios. The objective is to compare the severity of disruption to the SC performance of the different SC network solutions obtained in Sect. 4. We generated six independent disruptive scenarios, each representing a disruption in each region. Table 12 presents the list of suppliers, plants, and warehouses that were affected by a disruption in each scenario.

**Table 12:** Disrupted SC entities in each scenario

Scenario	Region	Affected suppliers		Affected plants		Affected warehouses	
		Bi-criterion SCN*	Profit SCN	Bi-criterion SCN	Profit SCN	Bi-criterion SCN	Profit SCN
1	Region 1	S16	S16	M3	M3	–	–
2	Region 2	S5, S8, S11	S5, S8, S11, S15	M1, M2	M1, M2	–	–
3	Region 3	S6, S7, S9	S6, S7, S9	M4	M4	W8	W8
4	Region 4	S4, S13	S4, S13	M5	M5	W12, W23	W12, W23
5	Region 5	S2	–	–	–	–	–
6	Region 6	–	–	–	–	–	–

^*^SCN SC network

In the MILP model, we set the binary variables relating to the disrupted entities to zero and re-optimized the model to measure the SC profit and unfulfilled demand. Note that the SC profit is the difference between the revenue and variable costs (purchasing cost, production cost, transportation cost, and lost sales cost). The fixed cost was not included in the profit calculation because the location decision had already been made. Tables 13 and 14 provide the revenue, variable costs, and unfulfilled demand associated with each disruptive scenario. Table 15 compares the SC profit of two SC network design solutions under disruptive scenarios.

**Table 13:** SC performance under disruptive scenarios (profit maximization model)

Scenario	Revenue ($)	Purchasing cost ($)	Production cost ($)	Transportation cost ($)	Lost sales cost ($)	Profit ($) (excluding fixed cost)	Unfilled demand in units (in %)
1	47,692,800.00	24,844,342.58	4,216,922.98	5,716,864.97	155,282.00	12,759,387.48	6572 (11)
2	34,882,200.00	18,176,671.72	2,838,619.48	4,146,147.81	517,106.00	9,203,654.99	20,806 (34.9)
3	35,895,600.00	18,422,882.57	3,107,821.31	4,322,184.21	491,899.00	9,550,812.91	19,680 (33)
4	17,658,900.00	9,101,720.95	1,320,737.06	2,148,856.82	1,077,029.00	4,010,556.17	39,943 (67.1)
5	53,554,500.00	27,784,596.65	4,468,797.16	6,390,310.71	1,298.00	14,909,497.48	59 (0.1)
6	53,554,500.00	27,784,596.65	4,468,797.16	6,390,310.71	1,298.00	14,909,497.48	59 (0.1)

**Table 14:** SC performance under disruptive scenarios (bi-criteria model)

Scenario	Revenue ($)	Purchasing cost ($)	Production cost ($)	Transportation cost ($)	Lost sales cost ($)	Profit ($) (excluding fixed cost)	Unfilled demand in units (in %)
1	48,753,900.00	25,440,020.85	4,314,265.12	5,846,582.71	127,558.00	13,025,473.32	5393 (9.1)
2	36,302,400.00	18,953,875.64	2,944,271.84	4,315,233.90	474,398.00	9,614,620.62	19,228 (32.3)
3	35,895,600.00	18,447,318.22	3,107,979.68	4,322,224.11	491,899.00	9,526,178.98	19,680 (33)
4	17,658,900.00	9,101,720.95	1,320,737.06	2,148,856.82	1,077,029.00	4,010,556.17	39,943 (67.1)
5	49,956,500.00	25,797,820.34	4,145,122.74	5,925,753.75	100,964.00	14,087,803.17	4279 (7.2)
6	53,607,600.00	27,753,548.34	4,460,868.95	6,408,485.96	-	14,884,886.49	59 (0.1)

**Table 15:** Comparison of the SC profit between two SC network design solutions under disruptive scenarios

Scenario	Profit SCN ($)	Bi-criteria SCN ($)	% Increase in profit
SC1	12,759,387.00	13,025,473.32	2.09
SC2	9,203,655.00	9,614,620.62	4.47
SC3	9,550,812.90	9,526,178.98	− 0.26
SC4	4,010,556.20	4,010,556.17	0.00
SC5	14,909,497.00	14,087,803.17	− 5.51
SC6	14,909,497.00	14,884,886.49	− 0.17
		Average % increase	0.10

Since the occurrence of the disruptive scenarios is different, we referred to the number of disasters, both natural and technological disasters reported by the Centre for Research on the Epidemiology of Disasters (Guha-Sapir et al., n.d.) between 1900 and 2014, to estimate the probability of disruption (see Table 16). The likelihood of occurrence for each scenario is the ratio between the number of disasters reported in a region and the total number of disasters reported. Table 16 shows the expected profit value and variance for each network design solution.

**Table 16:** Probability of occurrence for each scenario and expected SC profit under disruptive scenarios

Scenario	Number of disasters reported during 1900–2014	Probability of occurrence	Expected profit value of the profit maximization solution ($)	Expected profit value of the bi-criteria solution ($)
1	4345	0.21	12,759,387.48	13,025,473.32
2	8819	0.43	9,203,654.99	9,614,620.62
3	2736	0.13	9,550,812.91	9,526,178.98
4	2359	0.11	4,010,556.17	4,010,556.17
5	668	0.03	14,909,497.48	14,087,803.17
6	1646	0.08	14,909,497.48	14,884,886.49
Expected value			9,959,854.596	10,162,625.72
Variance			8.50173E+12	8.3124E+12

The expected profit of the SC network solution that was obtained from the profit maximization model is $9,959,864.60, with a variance of 8.5 × 10^12^. The expected payoff of the SC network solution that was obtained from the bi-criteria model is $10,162,625.72, with a variance of 8.31 × 10^12^. The high value of anticipated profit and the low value of profit variance both indicate that the SC network design solution obtained from the bi-criteria model is more resilient than the one obtained from the profit maximization model. The bi-criteria solution yielded a higher expected profit value of $202,771 or 2% than the profit maximization solution. The bi-criteria solution had a lower profit variance than the profit maximization solution (about 2.2%). Note that the cost of redundancy is $13,160,455–$12,880,839 = $279,615.68, as calculated from the difference in profit values between the profit maximization and the bi-criteria solutions.

### Discussions and managerial implications

In Sect. 5, we compared the SC performance of two SC network designs in a normal situation and under disruption. This section compares the number of selected suppliers of our model to that of Rienkhemaniyom and Pazhani (2015), as shown in Table 17.

**Table 17:** Comparison of the SC performance for different SC network designs with/without disruption

		SC network design model without maximum number of supplier constraint		SC network design model with the maximum number of suppliers = 10	
		Profit maximization	Bi-criteria model (*ɛ* = 0.68)	Profit maximization	Bi-criteria model (*ɛ* = 0.45)
No disruption	Number of selected suppliers	13	20	10	10
	profit value	$13,248,680	$12,954,399	$13,160,455.48	$12,880,839.75
	Density value	1.34	31.85	0.81	17.1
Under disruptions	Expected profit value	$10,602,016.36	$10,988,433.3	$9,959,854.59	$10,162,625.72
	variance of profit value	1.05E + 13	0.69E + 13	0.85E + 13	0.83E + 13
Mitigation cost		$294,281		$279,616	
Mitigation benefit		$386,417		$202,771	

The following are some of the inferences drawn from the analysis and the managerial implications from the model:

- SC network design, which is highly focused on profit maximization, tends to select suppliers that are clustered in regions that offer low costs. Even though the region has a relatively low possibility of facing random disruptions, our analysis shows that the expected profit loss and variance are high.
- The SC network design from the bi-criteria model diversifies risk by locating suppliers in different geographical locations. Even though it increases the probability of facing SC disruption, the expected profit loss, as well as its variance, is relatively low. Hence, the SC network design from the bi-criteria model is more resilient than that from the single-objective model. other.
- By adding a constraint to the model (limiting the maximum number of selected suppliers to 10), the SC profit decreases. In terms of resiliency, the SC network design from the bi-criteria model is more resilient than the one from the profit maximization model in both cases.
- Mitigation cost versus mitigation benefit: the mitigation benefit from the bi-criteria model (without the maximum number of supplier constraints) outweighs the mitigation cost. Conversely, the mitigation benefit from the bi-criteria model (with the maximum number of suppliers) is higher than the mitigation benefit from the profit maximization model. SC managers can use this comparison to evaluate the effectiveness of various mitigation strategies.

## Conclusions and future research

To manufacture and distribute products efficiently and effectively, network design in SC is one of the most crucial decisions to make. In this study, we attempted to integrate systemic risk theory and contingency theory to investigate the impact of supply density on SC resilience and the design of a four-stage SC network. Furthermore, the consideration of SCD and profitability in network design is novel, and this will open up new avenues for similar studies in the field.

We formulated a bi-criteria MILP model to optimally select suppliers, determine the location of facilities, and design a distribution plan between the selected set of facilities in order to maximize SC profit and supply density. We developed an interactive fuzzy solution approach based on the $\upvarepsilon$-constraint method to solve the proposed bi-criteria MILP model. The solution approach was able to interactively generate a Pareto-efficient frontier that represented a trade-off between SC profit and supply density objectives. A realistic illustrative example was solved to demonstrate the use of the bi-criteria MILP model and the interactive algorithm.

We also evaluated the resiliency of the SC network solutions and compared them based on the expected profit and variance. According to the findings, the SC network design that prioritized profit maximization tended to select facilities that were nearby such that the total cost was minimized. However, this resulted in a high expected severity of random disruptions. Conversely, the bi-criterion SC network design allowed redundancy in the SC by spreading facilities to different regions. Thus, the SC expected the disruptions to be less severe. For the illustrative example, the results indicate that the bi-criterion SC network design yielded a 2% higher expected profit and a 2.2% lower expected profit variance than the profit maximization network design. The model can help companies evaluate the trade-off between mitigation benefit and mitigation cost.

However, this study has limitations that allow scope for future work. First, the current model does not discuss the profit and risk-sharing mechanisms that govern the SC. We acknowledge that these have significant implications for sharing benefits and risks along the SC, and we believe that this can be considered as a follow-up study.

Second, the density of plants and warehouses in the network can be considered. In this case, the interstage and intrastage functions must also be extended to the other stages. We can also develop the mathematical model to incorporate other SC network characteristics, such as network complexity and node criticality, to gain a better understanding of SC resilience. The model can also be developed into a multiperiod model, and a risk profile can be formulated to evaluate SC resiliency on a tactical level.

Finally, the model does not consider the stochastic nature of the variables that impact SC profitability and resilience. Parameters such as demand and cost factors in the SC directly affect the network design and SC performance indicators. Based on its strategic nature, the problem can be extended by incorporating demand and cost uncertainty. Several researchers (Belen et al., 2009; Savku & Weber, 2017; Yılmaz et al., 2015) have investigated stochastical optimal control problems in diverse topics; such studies can also be conducted in the context of SC design and optimization.

## Appendix

See Tables

**Table 18:** Interstage distance (in miles) between the supplier and the manufacturing plants

	Plant 1	Plant 2	Plant 3	Plant 4	Plant 5
S1	12,143	9756	5466	5782	5338
S2	5502	6363	6417	8647	8803
S3	10,471	8038	4446	5816	5383
S4	7043	7154	7428	2322	2129
S5	560	2947	7380	5915	6351
S6	5498	4264	5331	4932	4945
S7	5170	4055	5488	4912	4965
S8	2824	525	4405	8454	8791
S9	5519	4113	5076	5189	5195
S10	1442	4221	8639	5060	5513
S11	720	3580	7945	5774	6226
S12	8696	9262	7812	2435	1983
S13	8270	9904	8897	1997	1607
S14	5351	4236	5496	4822	4855
S15	1977	2589	6747	5545	5907
S16	7856	5121	704	9699	9324
S17	1356	2101	6038	7711	8152
S18	4533	2579	4387	6461	6562
S19	5446	4620	5919	4344	4377
S20	7373	7790	7838	1907	1619

*S* supplier

18,

**Table 19:** Intrastage distance (in miles) between the suppliers

	S1	S2	S3	S4	S5	S6	S7	S8	S9	S10
S1	1,00,000	7076	1972	5164	11,636	6868	7190	9852	6865	10,714
S2	7076	1,00,000	8645	10,928	6037	10,626	10,389	5842	10,470	5797
S3	1972	8645	1,00,000	4207	10,041	5008	5340	8316	4966	10,400
S4	5164	10,928	4207	1,00,000	6484	3044	3168	7674	3264	6243
S5	11,636	6037	10,041	6484	1,00,000	5033	4701	2999	5077	1278
S6	6868	10,626	5008	3044	5033	1,00,000	333	4786	261	5688
S7	7190	10,389	5340	3168	4701	333	1,00,000	4580	471	5361
S8	9852	5842	8316	7674	2999	4786	4580	1,00,000	4631	4236
S9	6865	10,470	4966	3264	5077	261	471	4631	1,00,000	5801
S10	10,714	5797	10,400	6243	1278	5688	5361	4236	5801	1,00,000
S11	11,450	5514	10,686	6759	765	5706	5373	3543	5772	744
S12	3459	9185	3456	2130	8189	5031	5215	9787	5211	7482
S13	3873	8150	4452	2921	7871	5963	6084	10,367	6177	6876
S14	7004	10,579	5167	3011	4877	193	193	4760	421	5506
S15	10,346	7446	8538	5370	1503	3531	3200	2915	3573	2418
S16	4846	6226	4050	7502	8050	5791	5985	5057	5547	9290
S17	11,372	4878	10,129	8078	1805	5847	5556	1819	5780	2758
S18	7942	8909	5971	4788	4231	1752	1630	3087	1562	5288
S19	6876	10,868	5138	2572	4939	600	602	5145	862	5424
S20	4808	10,323	4163	664	6827	3708	3831	8305	3927	6395

	S11	S12	S13	S14	S15	S16	S17	S18	S19	S20
S1	11,450	3459	3873	7004	10,346	4846	11,372	7942	6876	4808
S2	5514	9185	8150	10,579	7446	6226	4878	8909	10,868	10,323
S3	10,686	3456	4452	5167	8538	4050	10,129	5971	5138	4163
S4	6759	2130	2921	3011	5370	7502	8078	4788	2572	664
S5	765	8189	7871	4877	1503	8050	1805	4231	4939	6827
S6	5706	5031	5963	193	3531	5791	5847	1752	600	3708
S7	5373	5215	6084	193	3200	5985	5556	1630	602	3831
S8	3543	9787	10,367	4760	2915	5057	1819	3087	5145	8305
S9	5772	5211	6177	421	3573	5547	5780	1562	862	3927
S10	744	7482	6876	5506	2418	9290	2758	5288	5424	6395
S11	1,00,000	8176	7618	5539	2214	8574	2014	4995	5548	6991
S12	8176	1,00,000	1226	5040	7374	7497	9973	6773	4643	1543
S13	7618	1226	1,00,000	5931	7513	8468	9624	7708	5484	2256
S14	5539	5040	5931	1,00,000	3379	5968	5750	1777	479	3675
S15	2214	7374	7513	3379	1,00,000	7451	2710	2869	3473	5854
S16	8574	7497	8468	5968	7451	1,00,000	6613	5019	6356	7807
S17	2014	9973	9624	5750	2710	6613	1,00,000	4432	5990	8526
S18	4995	6773	7708	1777	2869	5019	4432	1,00,000	2232	5452
S19	5548	4643	5484	479	3473	6356	5990	2232	1,00,000	3233
S20	6991	1543	2256	3675	5854	7807	8526	5452	3233	1,00,000

*S* supplier

19,

**Table 20:** The purchasing cost of the suppliers, the capacity of the suppliers, and the capacity of the manufacturing plants

	Plant 1	Plant 2	Plant 3	Plant 4	Plant 5	Capacity at supplier *i* (units)
S1	508.78	514.72	474.87	503.67	477.98	3969
S2	486.66	476.62	494.57	515.11	497.16	5483
S3	496.18	503.00	481.71	489.27	503.19	3113
S4	482.22	502.98	486.84	481.18	461.25	6295
S5	466.21	486.01	492.10	489.03	479.46	5975
S6	498.06	485.64	505.89	472.90	473.15	5788
S7	505.11	497.65	490.10	474.56	481.14	6900
S8	494.95	458.17	491.42	511.33	521.52	6882
S9	492.79	485.13	476.75	489.09	499.79	6990
S10	462.62	490.70	503.81	490.97	496.66	3767
S11	453.20	486.09	492.61	500.56	483.09	3670
S12	495.56	492.78	509.75	478.51	485.36	5646
S13	516.55	491.81	491.99	479.30	465.88	6188
S14	486.67	490.83	472.78	493.42	490.51	3604
S15	490.16	470.87	496.63	482.15	502.01	4304
S16	508.98	472.37	457.84	506.45	519.29	6597
S17	482.13	469.72	504.41	505.41	496.63	3453
S18	490.39	491.91	501.77	497.74	481.54	4538
S19	495.06	498.61	488.26	494.26	486.26	5057
S20	516.22	494.49	488.89	484.91	493.27	4578
Production cost at plant *m*	82.56	76.70	62.24	76.57	83.73	
Capacity of plant *m* (units)	17,442	18,262	16,478	11,029	12,829	

*S* supplier

20 and

**Table 21:** Capacity and fixed cost of the warehouses

	Size 1		Size 2		Size 3	
	Capacity (units)	Fixed cost	Capacity (units)	Fixed cost	Capacity (units)	Fixed cost
W1	10,704	429,710	13,972	484,574	20,815	599,472
W2	9221	404,814	16,831	532,581	18,571	561,788
W3	11,475	442,658	16,286	523,435	23,349	642,000
W4	8217	387,955	16,900	533,729	20,054	586,685
W5	7982	384,007	14,727	497,248	20,575	595,437
W6	8451	391,883	13,892	483,239	18,241	556,251
W7	6244	354,828	16,988	535,214	22,484	627,484
W8	10,030	418,406	15,965	518,043	19,621	579,420
W9	5969	350,216	17,592	545,354	22,075	620,613
W10	9303	406,197	14,467	492,889	21,678	613,955
W11	10,930	433,503	15,089	503,333	19,982	585,475
W12	8310	389,518	17,200	538,780	21,207	606,052
W13	6841	364,853	17,675	546,755	22,429	626,558
W14	10,844	432,061	15,275	506,460	21,700	614,329
W15	11,362	440,753	17,524	544,214	19,656	580,012
W16	7181	370,575	12,708	463,363	20,022	586,151
W17	7347	373,362	17,101	537,118	21,393	609,170
W18	8648	395,190	15,305	506,954	19,381	575,390
W19	9152	403,661	14,547	494,239	23,218	639,801
W20	7691	379,137	14,665	496,211	22,235	623,307
W21	6931	366,364	16,977	535,030	23,565	645,632
W22	10,637	428,585	12,036	452,082	21,585	612,395
W23	9518	409,799	13,224	472,028	18,677	563,570
W24	7578	377,227	15,985	518,368	19,037	569,619
W25	8287	389,135	17,718	547,476	18,519	560,910

*W* warehouse

21.

## References

[CR1] Anderson B (2007). Securing the supply chain-prevent cargo theft. Security.

[CR2] Araz C, Mizrak Ozfirat P, Ozkarahan I (2007). An integrated multicriteria decision-making methodology for outsourcing management. Computers and Operations Research.

[CR3] Aryanezhad M, Gholamreza S, Naini J, Jabbarzadeh A (2012). An integrated model for designing supply chain network under demand and supply uncertainty. African Journal of Business Management.

[CR4] Belen S, Kropat E, Weber G-W (2009). On the classical Maki-Thompson rumour model in continuous time. Central European Journal of Operations Research.

[CR5] Berman O, Krass D, Menezes M (2007). Facility reliability issues in network p-median problems: Strategic centralization and co-location effects. Operations Research.

[CR6] Bilsel R, Ravindran A (2011). A multiobjective chance constrained programming model for supplier selection under uncertainty. Transportation Research Part B: Methodological.

[CR7] Bode C, Wagner S (2015). Structural drivers of upstream supply chain complexity and the frequency of supply chain disruptions. Journal of Operations Management.

[CR8] Burns T, Stalker GM (1961). The management of innovation.

[CR9] Chai J, Liu J, Ngai E (2013). Application of decision-making techniques in supplier selection: A systematic review of literature. Expert Systems with Applications.

[CR10] Chan F, Jha A, Tiwari M (2016). Bi-objective optimization of three echelon supply chain involving truck selection and loading using NSGA-II with heuristics algorithm. Applied Soft Computing Journal.

[CR11] Cheraghalipour A, Paydar M, Hajiaghaei-Keshteli M (2018). A bi-objective optimization for citrus closed-loop supply chain using Pareto-based algorithms. Applied Soft Computing Journal.

[CR12] Chesbrough H (2020). To recover faster from Covid-19, open up: Managerial implications from an open innovation perspective. Industrial Marketing Management.

[CR13] Christopher M, Peck H, Towill D (2006). A taxonomy for selecting global supply chain strategies. The International Journal of Logistics Management.

[CR14] Cohen M, Lee H (2020). Designing the right global supply chain network. Manufacturing and Service Operations Management.

[CR15] Craighead C, Blackhurst J, Rungtusanatham M, Handfield R (2007). The severity of supply chain disruptions: Design characteristics and mitigation capabilities. Decision Sciences.

[CR16] Currie C, Fowler J, Kotiadis K, Monks T, Onggo B, Robertson D, Tako A (2020). How simulation modelling can help reduce the impact of COVID-19. Journal of Simulation.

[CR17] Dai Z, Dai HM (2016). Bi-objective closed-loop supply chain network design with risks in a fuzzy environment. Journal of Industrial and Production Engineering.

[CR18] Darestani S, Hemmati M (2019). Robust optimization of a bi-objective closed-loop supply chain network for perishable goods considering queue system. Computers and Industrial Engineering.

[CR19] De Boer L, Labro E, Morlacchi P (2001). A review of methods supporting supplier selection. European Journal of Purchasing and Supply Management.

[CR20] Ehrgott M (2005). Multicriteria optimization.

[CR21] Elsinger H, Lehar A, Summer M (2006). Risk assessment for banking systems. Management Science.

[CR22] Falasca, M., Zobel, C., & Cook, D. (2008). A decision support framework to assess supply chain resilience. In *Proceedings of the 5th international ISCRAM conference* (pp. 596–605).

[CR23] Farahani R, Rezapour S, Drezner T, Fallah S (2014). Competitive supply chain network design: An overview of classifications, models, solution techniques and applications. Omega (United Kingdom).

[CR24] Fazli-Khalaf M, Mirzazadeh A, Pishvaee MS (2017). A robust fuzzy stochastic programming model for the design of a reliable green closed-loop supply chain network. Human and Ecological Risk Assessment.

[CR25] Garcia-Herreros P, Wassick J, Grossmann I (2014). Design of resilient supply chains with risk of facility disruptions. Industrial and Engineering Chemistry Research.

[CR26] Goh M, Lim JYS, Meng F (2007). A stochastic model for risk management in global supply chain networks. European Journal of Operational Research.

[CR27] Goli A, Aazami A (2018). Accelerated cuckoo optimization algorithm for capacitated vehicle routing problem in competitive conditions. International Journal of Artificial Intelligence.

[CR28] Guha-Sapir, D., Below, R., & Hoyois, P. (n.d.). EM-DAT: International Disaster Database. www.emdat.be. Accessed 18 July 2018

[CR29] Harrison P, Houm P, Thomas D, Craighead C (2013). Supply chain disruptions are inevitable—get READI: Resiliency enhancement analysis via deletion and insertion. Transportation Journal.

[CR30] Ho W, Xu X, Dey P (2010). Multi-criteria decision making approaches for supplier evaluation and selection: A literature review. European Journal of Operational Research.

[CR31] Huang E, Goetschalckx M (2014). Strategic robust supply chain design based on the Pareto-optimal tradeoff between efficiency and risk. European Journal of Operational Research.

[CR32] Hwang C, Masud A (1979). Multiple objective decision making—methods and applications: A state-of-the-art survey.

[CR33] Irawan C, Jones D (2019). Formulation and solution of a two-stage capacitated facility location problem with multilevel capacities. Annals of Operations Research.

[CR34] Ivanov D, Das A, Choi T (2018). New flexibility drivers for manufacturing, supply chain and service operations. International Journal of Production Research.

[CR35] Ivanov D, Dolgui A (2020). Viability of intertwined supply networks: Extending the supply chain resilience angles towards survivability. A position paper motivated by COVID-19 outbreak. International Journal of Production Research.

[CR36] Jiang J, Wu D, Chen Y, Li K (2019). Complex network oriented artificial bee colony algorithm for global bi-objective optimization in three-echelon supply chain. Applied Soft Computing Journal.

[CR37] Keynes JM (1937). General theory of employment, interest and money. The Quarterly Journal of Economics.

[CR38] Khalilpourazari S, Arshadi Khamseh A (2019). Bi-objective emergency blood supply chain network design in earthquake considering earthquake magnitude: A comprehensive study with real world application. Annals of Operations Research.

[CR39] Khalilpourazari S, Soltanzadeh S, Weber GW, Roy SK (2020). Designing an efficient blood supply chain network in crisis: Neural learning, optimization and case study. Annals of Operations Research.

[CR40] Kim Y, Chen Y, Linderman K (2015). Supply network disruption and resilience: A network structural perspective. Journal of Operations Management.

[CR41] Klibi W, Martel A (2012). Scenario-based supply chain network risk modeling. European Journal of Operational Research.

[CR42] Latha Shankar B, Basavarajappa S, Kadadevaramath R, Chen J (2013). A bi-objective optimization of supply chain design and distribution operations using non-dominated sorting algorithm: A case study. Expert Systems with Applications.

[CR43] Mari S, Lee Y, Memon M (2014). Sustainable and resilient supply chain network design under disruption risks. Sustainability.

[CR44] McGillivray G (2000). Commercial risk under JIT. Canadian Underwriter.

[CR45] Meixell M, Gargeya V (2005). Global supply chain design: A literature review and critique. Transportation Research Part E: Logistics and Transportation Review.

[CR46] Melo MT, Nickel S, Saldanha-da-Gama F (2009). Facility location and supply chain management: A review. European Journal of Operational Research.

[CR47] Mintzberg H (1978). Patterns in strategy formation. Management Science.

[CR48] Nagurney A (2010). Supply chain network design under profit maximization and oligopolistic competition. Transportation Research Part E: Logistics and Transportation Review.

[CR49] Namdar J, Li X, Sawhney R, Pradhan N (2018). Supply chain resilience for single and multiple sourcing in the presence of disruption risks. International Journal of Production Research.

[CR50] Nikolopoulos, K., Punia, S., Schäfers, A., Tsinopoulos, C., & Vasilakis, C. (2020). Forecasting and planning during a pandemic: COVID-19 growth rates, supply chain disruptions, and governmental decisions. *European Journal of Operational Research*, In press. 10.1016/j.ejor.2020.08.001 PMC7413852 32836717

[CR51] Özceylan E, Paksoy T (2013). A mixed integer programming model for a closed-loop supply-chain network. International Journal of Production Research.

[CR52] Özceylan E, Paksoy T (2013). Fuzzy multi-objective linear programming approach for optimising a closed-loop supply chain network. International Journal of Production Research.

[CR53] Pazhani, S., Beeg, T., Kowalczyk, K., & Dietrich, T. (2018). A bi-criteria mixed integer linear programming model for load balancing and chemical saving in wafer cleaning processes: IE: Industrial engineering. In *2018 29th Annual SEMI Advanced Semiconductor Manufacturing Conference, ASMC* 2018 (pp. 49–53). Institute of Electrical and Electronics Engineers Inc.

[CR54] Pazhani S, Ravindran A (2014). Design of closed loop supply chain networks. International Journal of Business Analytics.

[CR55] Peck H (2005). Drivers of supply chain vulnerability: An integrated framework. International Journal of Physical Distribution & Logistics Management.

[CR56] Peng P, Snyder L, Lim A, Liu Z (2011). Reliable logistics networks design with facility disruptions. Transportation Research Part B: Methodological.

[CR57] Pervin M, Roy SK, Weber GW (2018). Analysis of inventory control model with shortage under time-dependent demand and time-varying holding cost including stochastic deterioration. Annals of Operations Research.

[CR58] Pinto-Varela T, Barbosa-Póvoa A, Novais A (2011). Bi-objective optimization approach to the design and planning of supply chains: Economic versus environmental performances. Computers and Chemical Engineering.

[CR59] Pishvaee M, Razmi J (2012). Environmental supply chain network design using multi-objective fuzzy mathematical programming. Applied Mathematical Modelling.

[CR60] Qi L, Shen Z (2007). A supply chain design model with unreliable supply. Naval Research Logistics.

[CR61] Ramezani M, Kimiagari AM, Karimi B, Hejazi TH (2014). Closed-loop supply chain network design under a fuzzy environment. Knowledge Based Systems.

[CR62] Ramkumar N, Subramanian P, Narendran T, Ganesh K (2012). Mixed integer linear programming model for multi-commodity multi-depot inventory routing problem. OPSEARCH.

[CR63] Ravindran, A. (2016). *Multiple criteria decision making in supply chain management* (1st ed.). CRC press. https://www.routledge.com/Multiple-Criteria-Decision-Making-in-Supply-Chain-Management/Ravindran/p/book/9781498708586. Accessed 27 August 2020

[CR64] Ravindran A, Bilsel U, Wadhwa V, Yang T (2010). Risk adjusted multicriteria supplier selection models with applications risk adjusted multicriteria supplier selection models with applications. International Journal of Production Research.

[CR65] Ravindran A, Warsing DJ (2016). Supply chain engineering: Models and applications.

[CR66] Rienkhemaniyom, K., & Pazhani, S. (2015). A Supply Chain Network Design Considering Network Density. In V. Kachitvichyanukul, K. Sethanan, & P. Golinska-Dawson (Eds.), *Toward sustainable operations of supply chain and logistics systems* (pp. 3–19). Springer, Cham.

[CR67] Sabri E, Beamon B (2000). A multi-objective approach to simultaneous strategic and operational planning in supply chain design. Omega.

[CR68] Salehi F, Mahootchi M, Husseini SMM (2017). Developing a robust stochastic model for designing a blood supply chain network in a crisis: A possible earthquake in Tehran. Annals of Operations Research.

[CR69] Sangaiah AK, Tirkolaee EB, Goli A, Dehnavi-Arani S (2020). Robust optimization and mixed-integer linear programming model for LNG supply chain planning problem. Soft Computing.

[CR70] Santoso T, Ahmed S, Goetschalckx M, Shapiro A (2005). A stochastic programming approach for supply chain network design under uncertainty. European Journal of Operational Research.

[CR71] Sarkis J, Cohen M, Dewick P, Schröder P (2020). A brave new world: Lessons from the COVID-19 pandemic for transitioning to sustainable supply and production. Resources, Conservation and Recycling..

[CR72] Savku E, Weber G-W (2017). A stochastic maximum principle for a Markov regime-switching jump-diffusion model with delay and an application to finance. Journal of Optimization Theory and Applications.

[CR73] Sawik T (2013). Integrated selection of suppliers and scheduling of customer orders in the presence of supply chain disruption risks. International Journal of Production Research.

[CR74] Sawik T (2014). Joint supplier selection and scheduling of customer orders under disruption risks: Single versus dual sourcing. Omega (United Kingdom).

[CR75] Scheibe KP, Blackhurst J (2018). Supply chain and information systems publications supply chain and information systems supply chain disruption propagation: A systemic risk and normal accident theory perspective. International Journal of Production Research.

[CR76] Schmitt, A., & Singh, M. (2009). Quantifying supply chain disruption risk using Monte Carlo and discrete-event simulation. In *Proceedings of the 2009 winter simulation conference (WSC)* (pp. 1237–1248). IEEE.

[CR77] Snyder L, Atan Z, Peng P, Rong Y, Schmitt A, Sinsoysal B (2016). OR/MS models for supply chain disruptions: A review. IIE Transactions (Institute of Industrial Engineers).

[CR78] Snyder L, Daskin M (2005). Reliability models for facility location: The expected failure cost case. Transportation Science.

[CR79] Squire, B. (2010). Managing supply chain risks: Understanding the impact of network characteristics. In S. Ponis (Eds.), *Managing Risk in Virtual Enterprise Networks: Implementing Supply Chain Principles* (pp. 28–48). IGI Global.

[CR80] Stecke K, Kumar S (2009). Sources of supply chain disruptions, factors that breed vulnerability, and mitigating strategies. Journal of Marketing Channels.

[CR81] Turkoglu D, Genevois M (2020). A comparative survey of service facility location problems. Annals of Operations Research.

[CR82] Wachtendorf T, Brown B, Holguin-Veras J (2013). Catastrophe characteristics and their impact on critical supply chains: Problematizing materiel convergence and management following hurricane katrina. Journal of Homeland Security and Emergency Management.

[CR83] Wagner S, Bode C (2006). An empirical investigation into supply chain vulnerability. Journal of Purchasing and Supply Management.

[CR84] Wagner S, Neshat N (2010). Assessing the vulnerability of supply chains using graph theory. International Journal of Production Economics.

[CR85] Xia W, Wu Z (2007). Supplier selection with multiple criteria in volume discount environments. Omega.

[CR86] Yılmaz F, Haceröz HH, Weber G-W (2015). Simulation of stochastic optimal control problems with symplectic partitioned Runge-Kutta scheme. Dynamics of Continuous, Discrete and Impulsive Systems: Series B.

